# The new Primary Care and Risk Factor Management (PCRFM) nucleus of the European Association of Preventive Cardiology: A call for action

**DOI:** 10.1177/2047487319894107

**Published:** 2019-12-16

**Authors:** Monika Hollander, Christi Deaton, Irene Gibson, Donata Kurpas, Frans Rutten, Henner Hanssen, Maria Antonopoulou, Paul Dendale, Diederick E Grobbee, on behalf of all PCRFM nucleus members

**Affiliations:** 1 Julius Centre for Health Sciences and Primary Care, University Medical Centre Utrecht, University of Utrecht, The Netherlands; 2 University of Cambridge School of Clinical Medicine, Department of Public Health and Primary Care, UK; 3 Cambridge University Hospitals NHS Foundation Trust, Cambridge Biomedical Campus, UK; 4 National Institute for Prevention and Cardiovascular Health, Galway, Ireland; 5 Department of Family Medicine, Wrocław Medical University, Poland; 6 Opole Medical School, Poland; 7 Department of Sport, Exercise and Health, University of Basel, Switzerland; 8 Spili Primary Care Centre, Regional Health System of Crete, Greece; 9 Jessa Hospital, Heart Centre Hasselt, Belgium; 10 BIOMED – Biomedical Research Centre, Hasselt University, Belgium

In recent decades, cardiovascular mortality has reduced significantly. Among others, improved treatment options for cardiovascular disease (CVD) and a reduction of smoking since the 1960s have contributed to this decline.^1^ In many countries, smoking in public areas is prohibited. However, western dietary habits, including foods high in sugar, salt and fat, and lack of exercise are still persistent, leading to obesity, diabetes and hypertension. Yet, despite numerous guidelines on prevention and treatment of CVD with medication and lifestyle management, the incidence of CVD is still increasing in many countries, fuelled by rising obesity levels, sedentary lifestyles and increased longevity.^2^ In particular in the southern hemisphere CVD is on the increase, with high rates of obesity, diabetes and hypertension.^3^ Demographics are changing in low and middle income countries, fuelling the rise of chronic diseases and a persistent burden of infectious diseases overwhelming the limited health care resources.^4^ In western countries survival after CVD events has successfully improved; however, at the price of more patients living with chronic CVDs.^5^ These developments will lead to an increased demand on healthcare services now and in the years ahead for both the prevention and the management of CVD. The organisation and continuity of care through the various layers of the different national healthcare systems is challenging and calls for close collaboration between hospital specialists, general practitioners (GPs) and other primary care workers in the battle against CVD. GPs are key in identifying patients at risk of CVD and providing individualised, risk stratified preventive care.

GPs, primary care nurses and other members of the primary care team have longstanding relationships with patients from cradle to grave and have frequent contacts with those enlisted. In adults, CVD or risk factors are in the top 10 of reasons to consult the general practice.^6^ Thus, primary care professionals are in general the first to identify changes in patients’ CVD risk factors. The 2016 European Guidelines on cardiovascular disease prevention in clinical practice recommend the prevention of CVD to be delivered in all healthcare settings, including primary care.^7^ It further recommends that GPs, nurses and allied health professionals such as physiotherapists, dieticians and psychologists should work together as a team to provide the most effective multidisciplinary care. General practice can offer comprehensive management for patients with CVD, including those with multiple comorbid conditions. Nevertheless, more implementation of individualised risk-based prevention that focuses on all aspects of lifestyle and risk factor management is needed. Important in this perspective of multi-morbidity is the risk of overtreatment and conflicting recommendations from different guidelines.

There is a large variation in the organisation of primary care within Europe and even more so globally.^8^ Consequently, the organisation of integrated cardiovascular risk management (CVRM) will show large variations, depending on factors such as reimbursement, organisation of national healthcare systems, for example, GP as gate keeper to hospital specialists, availability of international guidelines translated into the native language and adapted to the regional situation.

In some EU countries with a strong primary care system and reimbursement for cardiovascular risk management care, integrated cardiovascular risk programmes for high CVD risk patients have already been implemented, starting with the point that patients receive the ‘right care at the right place, in secondary care if necessary and primary care in collaboration with specialists if possible’.^9^ For this purpose, detailed regional agreements are applied between hospital specialists and general practices, focused on substitution of care from hospital to near home setting. The effectiveness of such care is still under investigation.

The EUROASPIRE studies consistently show that the proportion of high CVD risk patients actually reaching treatment goals is modestly improving over years, but it is still disappointingly low.^10^ A large majority of patients are not reaching the recommended lifestyle and therapeutic goals for blood pressure, lipids and glucose and furthermore only a minority of patients are being advised to participate in CVD prevention programmes. Guidelines on management of CVD and risk factors are very clear on these goals, but implementation in practice is difficult. More detailed knowledge is needed to identify barriers for implementation and possible solutions to provide better patient centred CVD preventive care.

To address above mentioned challenges, the European Association of Preventive Cardiology (EAPC) has launched a new nucleus ‘Primary Care and Risk Factor Management’ (PCRFM). The aims are: (i) investigating daily practice, (ii) defining standards and (iii) promoting CVD prevention in primary care, including risk-stratified management for those at risk and with already established cardiovascular disease.”

The full nucleus was established in February 2019 and members come from different disciplines across primary and secondary care, all with strong links to primary care; for example, GPs, cardiologists, vascular internists, occupational health specialists, nurses, physiotherapists, psychologists, dieticians and exercise physiologists.

The PCRFM has committed itself to reaching the following goals in the coming years:

- Establishing itself as an entity within and outside of the European Association of Preventive Cardiology, reaching out to and seeking collaboration with all relevant stakeholders in specialist and primary care groups involved in CVD prevention;
- Building a strong network for all disciplines working in primary care, taking joint action to act as advocates to improve the standards of CVD prevention in primary care across Europe;
- To help build stronger primary care systems in countries where this is a prerequisite for adequate prevention and treatment of CVD;
- Contributing to scientific sessions and tracks in European Society of Cardiology (ESC) conferences related to multidisciplinary primary CVRM care and the role of general practice in general CVD prevention;
- Providing position papers that synthesise evidence relating to risk factor management and delivery of prevention in primary care and other settings, including the community and workplace;
- Commenting on ESC guidelines with regard to implications for primary care, ‘How to’ and contributing to the development of ESC practice guidelines;
- Developing educational activities in cooperation with relevant partners for the wide scope of professionals working in primary care and the community;
- Initiating and contributing to scientific research projects related to the defined goals.

The PCRFM has a unique position to act as liaison between various disciplines from primary care and the different bodies within the ESC and specialist care. The PCRFM can add value with regard to important themes for primary care such as, for example, how to reach the high-risk population, how to deal with multimorbidity and how to reach out to the lower income populations. The launch of the PCRFM section within the EAPC is a consequence of the growing needs for a multidisciplinary, patient-centred, individualised approach for CVD in primary care settings to meet the growing socio-economic healthcare challenges of modern society. The section will promote primary care and risk factor management in Europe and beyond, facilitating an international network of primary care professionals with a focus on education, research and clinical guideline implementation.

*https://www.escardio.org/Sub-specialty-communities/European-Association-of-Preventive-Cardiology-(EAPC)/About/primary-care-and-risk-factor-management-section*
